# Retraction: Analysis of psychological characteristics and emotional expression based on deep learning in higher vocational music education

**DOI:** 10.3389/fpsyg.2024.1433717

**Published:** 2024-05-27

**Authors:** Frontiers Editorial Office

The Journal retracts the 2022 article cited above.

Following publication, the publisher found evidence of peer review manipulation. As the scientific integrity of the article cannot be guaranteed, and in adherence to the recommendations of the Committee on Publication Ethics (COPE), the article is retracted. This retraction was approved by the Chief Executive Editor of Frontiers. The authors have not responded to correspondence regarding this retraction.

